# Development of variously functionalized nitrile oxides

**DOI:** 10.3762/bjoc.11.138

**Published:** 2015-07-23

**Authors:** Haruyasu Asahara, Keita Arikiyo, Nagatoshi Nishiwaki

**Affiliations:** 1 School of Environmental Science and Engineering, Kochi University of Technology, Tosayamada, Kami, Kochi 782-8502, Japan; 2 Research Center for Material Science and Engineering, Kochi University of Technology, Tosayamada, Kami, Kochi 782-8502, Japan

**Keywords:** acylnitrile oxide, amide, formylnitrile oxide, functionalized nitrile oxide, Weinreb amide

## Abstract

*N*-Methylated amides (*N*,4-dimethylbenzamide and *N*-methylcyclohexanecarboxamide) were systematically subjected to chemical transformations, namely, *N*-tosylation followed by nucleophilic substitution. The amide function was converted to the corresponding carboxylic acid, esters, amides, aldehyde, and ketone upon treatment with hydroxide, alkoxide, amine, diisobutylaluminium hydride and Grignard reagent, respectively. In these transformations, *N*-methyl-*N*-tosylcarboxamides behave like a Weinreb amide. Similarly, *N*-methyl-5-phenylisoxazole-3-carboxamide was converted into 3-functionalized isoxazole derivatives. Since the amide was prepared by the cycloaddition reaction of ethynylbenzene and *N*-methylcarbamoylnitrile oxide, the nitrile oxide served as the equivalent of the nitrile oxides bearing a variety of functional groups such as carboxy, alkoxycarbonyl, carbamoyl, acyl and formyl moieties.

## Introduction

Nitrile oxides are valuable synthetic synthons for the construction of heterocyclic compounds by cycloaddition reactions, which lead to the formation of two bonds in a single experimental step [1–2]. In addition, cycloadducts serve as precursors of polyfunctionalized compounds by ring opening reaction followed by N–O bond fission [2–3]. Hence, functionalized nitrile oxides are clearly useful for the preparation of more complex systems. However, because the precursors of the functionalized nitrile oxides are not always easily available, there are only a few reports that deal with these compounds compared to those on alkylated and arylated nitrile oxides [1]. Thus, it is highly desirable to develop methodologies for the facile generation of functionalized nitrile oxides.

**Scheme 1:**
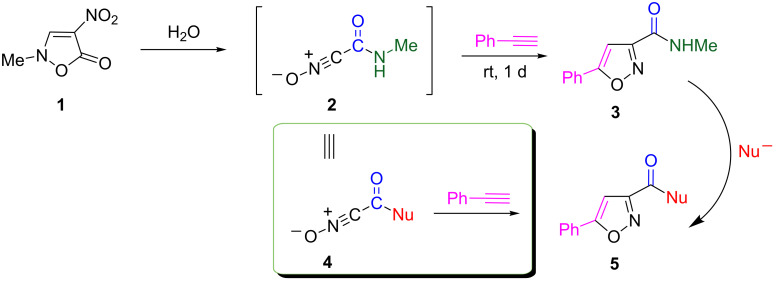
A strategy for developing functionalized nitrile oxides.

In our previous work, we reported the preparation of nitrile oxide **2** bearing an *N*-methylcarbamoyl group by treatment of 2-methyl-4-nitroisoxazolin-5(2*H*)-one (**1**) only with water at room temperature. Nitrile oxide **2** undergoes cycloaddition reactions with alkenes [4–5], alkynes (Scheme 1) [4–5], nitriles [6], and 1,3-dicarbonyl compounds [7] to afford the corresponding polyfunctionalized heterocyclic compounds, which are not easily obtained by other approaches. Although this protocol is expected to be useful for synthesizing polyfunctionalized compounds, it is limited by the need of a *N*-methylcarbamoyl functional group. The synthetic utility of nitrile oxide **2** will be indeed improved by converting the *N*-methylcarbamoyl group into other functionalities, i.e., nitrile oxide **2** may serve as an equivalent of nitrile oxides **4** having versatile functional groups (Scheme 1).

Weinreb amide (*N*-methoxy-*N*-methylamide) is widely used for the conversion of less reactive amide moieties into other carbonyl functionalities [8–9]. The two oxygen atoms of the methoxy and carbonyl groups coordinate to the organometallic reagents, thereby suppressing further reaction with the obtained aldehyde or ketone. However, this protocol cannot be applied to the conversion of *N*-methylamides because methoxylation of *N*-methylamides is hitherto unknown. Thus, we speculated that the introduction of a sulfonyl group would be effective because of its high electron-withdrawing and coordinating abilities, serving as an equivalent of the Weinreb amide. Indeed, the use of *N*-methyl-3-phenyl-*N*-tosylbutanamide for this purpose was already reported by Itoh and coworkers [10]. However, a systematic study using simple amides has not yet been performed. In this context, we examined the sulfonylation and chemical conversion of *N*-methylated aromatic and aliphatic amides, and a similar conversion of *N*-methyl-5-phenylisoxazole-3-carboxamide (**3**), which is equal to the generation of variously functionalized nitrile oxides.

## Results and Discussion

At the outset, methanesulfonylation (mesylation) of amides was studied. To a suspension of *N*,4-dimethylbenzamide (**6A**) and sodium hydride (2 equiv) in THF, mesyl chloride (**7a**, 1.3 equiv) was added, and the resultant mixture was stirred at room temperature for 9 h. After acidification, the reaction mixture was extracted with diethyl ether to afford the crude material, from which 32% of *N*-mesylated product **8Aa** [11] was isolated and 61% of **6A** was recovered. Thus, the conversion yield of **8Aa** was 82% (Table 1, entry 1). On the other hand, the yield of **8Aa** was considerably increased (up to 70%) when ethylmagnesium bromide was employed as a base instead of sodium hydride (Table 1, entry 2). However, the instability and gradual decomposition of mesylated product **8Aa** by ambient moisture impedes its storage and use as a synthetic reagent. Hence, in the place of the mesyl group, the 4-methylbenzenesulfonyl (tosyl) moiety was employed as the activating group of the amide function. Indeed, tosylated product **8Ab** [11] proved to be sufficiently stable under ambient conditions. Despite the considerable efforts for optimizing the reaction conditions, the highest yield obtained for **8Ab** was 60%; however, 35% of starting material **6A** was recovered, and the conversion yield was 92%. This tosylation method was applied to the aliphatic amide *N*-methylcyclohexanecarboxamide (**6B**) to afford **8Bb** upon treatment with tosyl chloride (**7b**) using sodium hydride as a base (Table 1, entry 4). In this case, the use of an excess of **7b** increased the yield of **8Bb** up to 60% (85% of conversion yield, Table 1, entry 5).

**Table 1:** Sulfonylation of *N*-methylamides.

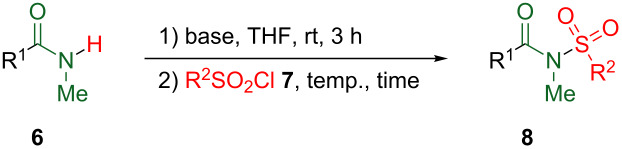							

entry	R^1^	R^2^	Base (equiv)	Temp./°C	Time/h	Product	Yield/%

1	4-MeC_6_H_4_	Me	NaH (2)	rt	9	**8Aa**	32 (82)^a^
2	4-MeC_6_H_4_	Me	EtMgBr (1.1)	0	1	**8Aa**	70
3	4-MeC_6_H_4_	4-MeC_6_H_4_	NaH (2)	0	24	**8Ab**	60 (92)^a^
4	*c*-Hexyl	4-MeC_6_H_4_	NaH (2)	0	12	**8Bb**	38
5^b^	*c*-Hexyl	4-MeC_6_H_4_	NaH (2)	0	12	**8Bb**	60 (85)^a^

^a^Conversion yields were shown in parentheses; ^b^5 equivalents of **7b** were used.

Next, nucleophilic substitutions of tosylated amides **8Ab** and **8Bb** were investigated (Table 2). Whereas water and methanol did not alter the amide moieties, hydroxide and methoxide converted amides **8Ab** and **8Bb** into the corresponding carboxylic acids (**9a** and **10a**) and methyl esters (**9b** and **10b**), respectively (Table 2, entries 1–4). In addition, the nucleophilic substitution also proceeded by employing neutral amines. The reaction of **8** with propylamine proceed smoothly, leading to *N*-propylamides **9c** and **10c** (Table 2, entries 5 and 6). The substitution reaction was influenced by the bulkiness of the amines. Heating at 120 °C was required for the substitution reaction by *sec*-butylamine. However, in the case of *tert*-butylamine, even when the reaction was conducted in a sealed tube at 120 °C, only a small amount of amide was observed (Table 2, entries 7–10). On the other hand, a cyclic secondary amine, pyrrolidine, afforded amides **9f** and **10f** quantitatively at room temperature (Table 2, entries 11 and 12).

**Table 2:** Chemical conversions of *N*-methylamide to other functions.

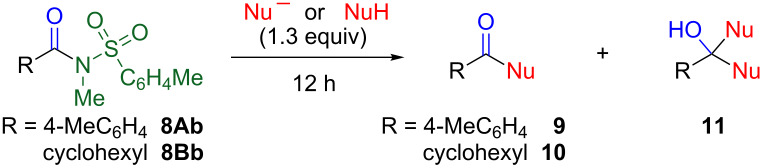						

entry	Amide	Nucleophile	Solvent	Temp./°C	Product	Yield/%

1	**8Ab**	NaOH	H_2_O	85	**9a**	quant.
2	**8Bb**	NaOH	H_2_O	85	**10a**	94
3	**8Ab**	NaOMe	MeOH	60	**9b**	62
4	**8Bb**	NaOMe	MeOH	60	**10b**	73
5	**8Ab**	PrNH_2_	THF	rt	**9c** [12]	quant.
6	**8Bb**	PrNH_2_	THF	rt	**10c**	82
7	**8Ab**	*sec*-BuNH_2_	THF	120	**9d** [13]	72
8	**8Bb**	*sec*-BuNH_2_	THF	120	**10d**	79
9	**8Ab**	*tert*-BuNH_2_	THF	120	**9e** [14]	19
10	**8Bb**	*tert*-BuNH_2_	THF	120	**10e** [15]	3
11	**8Ab**	pyrrolidine	THF	rt	**9f** [16]	quant.
12	**8Bb**	pyrrolidine	THF	rt	**10f** [17]	quant.
13	**8Ab**	BuLi	THF	−78	**9g**	0 (51)^a^
14	**8Ab**	iPrMgBr	THF	−40	**9h**	0 (58)^a^
15	**8Ab**	4-MeOC_6_H_4_MgBr	THF	−40	**9i** [18]	59
16	**8Bb**	4-MeOC_6_H_4_MgBr	THF	−40	**10i** [19]	33
17	**8Ab**	NaBH_4_	THF	−78	**9j**	0 (64)^a^
18	**8Ab**	iBu_2_AlH	THF	−78	**9j**	77 (12)^a^
19	**8Bb**	iBu_2_AlH	THF	−78	**10j**	39

^a^Intermediately produced **9** or **10** underwent the excess reactions to afford alcohols **11**.

When more reactive butyllithium or isopropylmagnesium bromide was used as the nucleophile, the corresponding tertiary alcohols **11g** and **11h** were obtained in 51% and 58% yields, respectively, whereas ketones **9g** and **9h** were not detected (Table 2, entries 13 and 14). In the case of an aromatic Grignard reagent, the undesired reaction was suppressed. Although the reaction did not occur at −78 °C, the substitution proceeded successfully at −40 °C, affording ketones **9i** and **10i** (Table 2, entries 15 and 16). Sodium borohydride also proved to be highly reactive, causing a further addition reaction even at −78 °C to generate the corresponding alcohol **11j** (Table 2, entry 17). This disadvantage was overcome by using bulkier diisobutylaluminium hydride (DIBAL) to furnish aldehydes **9j** and **10j**, although small amounts of alcohol **11j** were also detected (Table 2, entries 18 and 19).

In contrast to tosylated amides **8Ab** and **8Bb**, tosylated *N*-methyl-5-phenylisoxazole-3-carboxamide **12** exhibited higher reactivity. Upon tosylation under similar conditions **3** did not afford product **12**, and isoxazole-3-carboxylic acid **5a**, a hydrolyzed product of **12**, was observed. This problem was solved by quenching the reaction mixture with ice water instead of just water; yet, **12** was afforded in only 10% yield, and **5a** was the main product in 38% yield. These findings imply that during the work-up procedure, further decomposition of **12** by water occurred even at low temperatures because of the higher electron deficiency of the isoxazole ring compared with carbocyclic rings. When, instead of water, less nucleophilic acetic acid was employed for the quench, **12** was isolated in 74% yield and the competitive hydrolysis of **12** was suppressed (6% of **5a**).

Although tosylated amide **12** could be isolated, it gradually decomposed under ambient conditions. Hence, the chemical conversion was performed by adding a nucleophile to the mixture of the tosylation reaction, without the isolation of **12** (Table 3). As mentioned above, the use of a hydroxide anion for the hydrolysis was not required, and carboxylic acid **5a** was obtained in 81% yield by the addition of only water (Table 3, entry 1). This protocol was applicable to a relatively bulky alcohol to afford isopropyl ester **5k** in moderate yield (Table 3, entry 2). Similarly, primary and secondary amines underwent the substitution reaction, leading to **5c–f** in good to moderate yields (Table 3, entries 3–6). It was noteworthy that, because of high reactivity of **12** caused by the isoxazole ring [20], the methylamino group could be replaced by a bulky *tert*-butylamino moiety. Conversion of the *N*-methylcarbamoyl group to an acyl or formyl group was achieved upon treatment with a Grignard reagent or with DIBAL (Table 3, entries 7 and 8).

**Table 3:** Chemical conversion of *N*-methylisoxazole-3-carboxamide **3**.

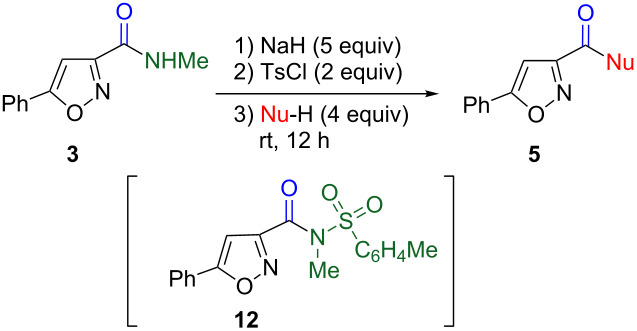			

entry	Nu-H		Yield/%

1	OH_2_	**5a** [21]	81
2	iPrOH	**5k** [22]	59
3	PrNH_2_	**5c** [23]	74
4	*sec*-BuNH_2_	**5d** [22]	59
5	*tert*-BuNH_2_	**5e** [24]	30
6	pyrrolidine	**5f** [23]	42
7^a,b^	4-MeOC_6_H_4_MgBr	**5i** [25]	45
8^a,c^	iBu_2_AlH	**5j** [26]	40

^a^2 Equiv of nucleophiles were used. ^b^At −40 °C. ^c^At −78°C.

**Figure 1:**
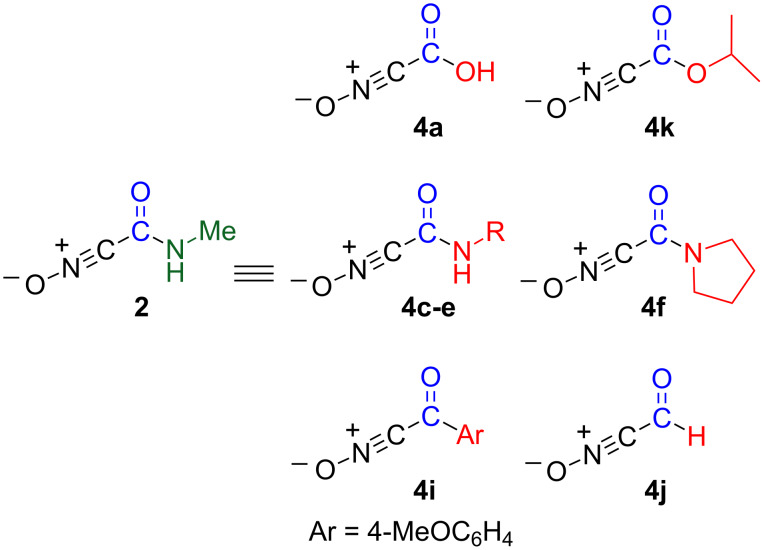
Versatile functionalized nitrile oxides.

The chemical conversion of *N*-methylamides to versatile carbonyl functions was systematically studied. In this protocol, the tosyl group was found to be effective for the activation of the carbamoyl group and for preventing over-addition by organometallic reagents. Isoxazole-3-carboxamide **3** was successfully converted in a similar fashion. Thus, as shown in Figure 1, nitrile oxide **2** serves as an equivalent of functionalized nitrile oxides **4**, thereby improving the synthetic utility of carbamoylnitrile oxide **2**.

## Experimental

### Conversion of isoxazolecarboxamide **3** to **5c** via tosyl derivative **12**

To a solution of amide **3** (101 mg, 0.5 mmol) in THF (1 mL), a suspension of 60 wt % sodium hydride (100 mg, 2.5 mmol) in THF (3 mL) was added under argon. After the mixture was stirred vigorously for 10 min, the mixture was cooled to 0 °C and then a solution of tosyl chloride (191 mg, 1 mmol) in THF (1 mL) was slowly added. After the mixture was stirred for 3 h at 0 °C, propylamine (164 μL, 2 mmol) was added, and then the mixture was stirred at room temperature for 12 h. After evaporation of the solvent, the residue was dissolved into diethyl ether (5 mL), washed with water (5 mL), and the aqueous layer was extracted with diethyl ether (2 × 5 mL). The combined organic layer was dried over magnesium sulfate, and concentrated, and the residue was subjected to column chromatography on silica gel to afford *N*-propylcarboxamide **5c** (85 mg, 0.37 mol, 74% yield), eluted with dichloromethane. ^1^H NMR (400 MHz, CDCl_3_, TMS) δ 1.01 (t, *J* = 7.2 Hz, 3H), 1.67 (tq, *J* = 7.2, 7.2 Hz, 2H), 3.43 (dt, *J* = 7.2, 7.2 Hz, 2H), 6.79–6.90 (br, 1H), 6.97 (s, 1H), 7.47–7.50 (m, 3H), 7.78–7.81 (m, 2H); ^13^C NMR (100 MHz, CDCl_3_, TMS) δ 11.4 (CH_3_), 22.7 (CH_2_), 41.2 (CH_2_), 99.2 (CH), 126.0 (CH), 126.9 (C), 129.1 (CH), 130.7 (CH), 158.9 (C), 159.3 (C), 171.5 (C).
